# Norbornene and Related Structures as Scaffolds in the Search for New Cancer Treatments

**DOI:** 10.3390/ph15121465

**Published:** 2022-11-25

**Authors:** Gorka Calvo-Martín, Daniel Plano, Nuria Martínez-Sáez, Carlos Aydillo, Esther Moreno, Socorro Espuelas, Carmen Sanmartín

**Affiliations:** 1 Departamento de Tecnología y Química Farmacéuticas, Universidad de Navarra, Irunlarrea 1, E-31008 Pamplona, Spain; 2 Instituto de Investigación Sanitaria de Navarra (IdiSNA), Irunlarrea, 3, E-31008 Pamplona, Spain; 3 Instituto de Salud Tropical, Universidad de Navarra, Irunlarrea 1, E-31008 Pamplona, Spain

**Keywords:** norbornene, norcantharidin, structural modulation, antitumoral activity, cancer therapy, drug delivery systems

## Abstract

The norbornene scaffold has arisen as a promising structure in medicinal chemistry due to its possible therapeutic application in cancer treatment. The development of norbornene-based derivatives as potential chemotherapeutic agents is attracting significant attention. Here, we report an unprecedented review on the recent advances of investigations into the antitumoral efficacy of different compounds, including the abovementioned bicyclic scaffold in their structure, in combination with chemotherapeutic agents or forming metal complexes. The impact that structural modifications to these bicyclic compounds have on the antitumoral properties and the mechanisms by which these norbornene derivatives act are discussed in this review. In addition, the use of norbornene, and its related compounds, encapsulation in nanosystems for its use in cancer therapies is here detailed.

## 1. Introduction

At present, cancer is the second leading cause of premature death worldwide. The International Agency for Research on Cancer (IARC) estimated that 19.3 million new cancer patients were reported in 2020, and the global cancer burden is expected to be 28.4 million cases in 2040 [1]. Despite the development of several cytotoxic agents, their clinical use is still very limited due to the appearance of undesirable side effects, such as toxicity and the development of resistance to treatment [2]. Even though targeted therapies have gained interest as cancer treatments [3], chemotherapeutic agents continue to play an important role in cancer pharmacotherapy. Currently, norbornene derivatives have attracted great attention from the scientific community due to their possible therapeutic applications in cancer treatment. Due to norbornene’s fast reactivity, it forms polymers that can be used as chemotherapeutic agent carriers [4] or in theranostic applications [5]. In addition, as will be discussed later in this review, a wide range of recent works have revealed interesting antitumoral properties concerning this molecule and its derivatives.

**Figure 1:**
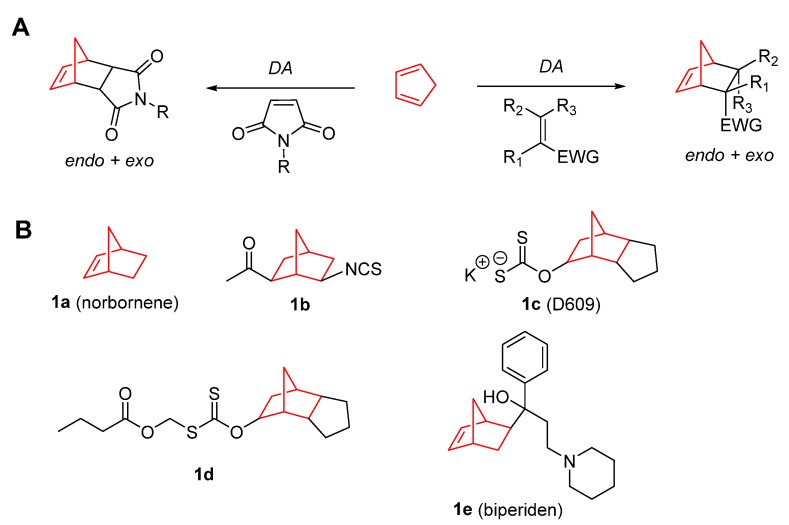
(**A**) Main synthetic routes for norbornene derivatives (EWG, electron-withdrawing group). (**B**) Norbornene and several other derivatives with antitumoral properties [19,20,21,22,23,24,25,26,27].

Norbornene (bicyclo[2.2.1]hept-2-ene) (**1a**) derivatives are active compounds derived from the Diels–Alder (DA) cycloaddition between cyclopentadiene and different dienophiles (Figure 1A). Due to the spontaneous dimerization of cyclopentadiene, it must be produced in situ after cracking the dicyclopentadiene by distillation, and should be used readily [6,7]. Thus, it reacts quickly and efficiently with activated dienophiles such as maleic anhydride and maleimides [8,9]. This unique reactivity provides norbornene and its derivatives with vast possibilities of chemical modification. As it has been previously mentioned, one of the main uses of norbornene is in polymer chemistry, where its polymerization is the most versatile among the cycloolefin addition polymerization mechanisms [10]. Norbornene can also be part of copolymers such as ethene–norbornene [11] or styrene–norbornene [12] copolymers. In addition, the double bond of this bicyclic structure produces greater ring strain, which can be released, turning it into a highly reactive alkene [13]. This feature makes the norbornene double bond especially reactive with sulfur species [14,15], which is a very useful tool for labeling cysteine [16]. Moreover, this bicyclic structure has been widely employed in biochemistry for the biorthogonal labeling of proteins and other biologically relevant molecules, taking advantage of its reactivity with tetrazines through an inverse electron demand DA reaction [16,17].

In addition, norbornene and related bicyclo[2.2.1] analogues, along with adamantane, have become the most exploited polycyclic structures as non-aromatic and non-planar motifs in drug design, in attempts to introduce variations in the so-called “flatland drugs” [18]. The preponderance of bicyclo[2.2.1] among all polycyclic motifs is mainly due to its abovementioned chemical accessibility through the Diels–Alder reaction.

Norbornene’s particular reactivity, together with the biological properties that this entity confers on other molecules, has drawn the attention of researchers during the last few decades, especially in the field of medicinal chemistry and cancer research. In this sense, this review provides an overview of the state of the art research on norbornene and its derivatives, with special focus on the antitumoral properties and therapeutic applications of these molecules.

## 2. Therapeutic Potential of Norbornene Derivatives in Cancer

A variety of norbornene derivatives with different substituents around its scaffold have been investigated as potential anticancer agents. Figure 1B shows the structures of norbornene and some norbornene derivatives that could potentially be employed for cancer treatment. The first study on the use norbornene derivatives was performed in 1994 using norbornyl isothiocyanates (**1b**), whose antitumoral properties where tested in mammary tumors being able to significantly reduce their size and delay tumor progression [19]. In 1997, Porn-Ares et al. [20] described the compound D609 (tricyclodecane-9-yl-xanthogenate, **1c**) as an apoptosis inducer in human monocytic leukemia cells (U937) through sphingomyelin synthase. The apoptosis induced by **1c** has also been associated with the level of cyclic adenosine monophosphate (cAMP) and p53 expression [21]. Additionally, it was able to increase ceramide and enhance rapidly induced apoptosis [22]. Moreover, further studies on D609 performed by Kalluri et al. [23] showed that this compound can induce cell death, with a concomitant decrease in GADD45β expression and an increase in the phosphorylation of p38 MAP kinase in glioma stem-like cells. Later, the same authors performed a study involving a chronic treatment with D609, reporting that not only the expression of GADD45β was decreased, but also cyclin-D1 and Olig2, two proteins that impede the progression of the cell cycle and curtail the growth of glioma stem-like cells [24]. However, despite its promising antitumor properties, D609 displays moderate antitumor activity in vivo. Another limitation of **1c** is that it can readily oxidize to form a disulfide bond, with the subsequent loss of its biological activities. In this sense, Bai et al. [25] proposed the inclusion of *S*-(alkoxyacyl) groups to form a prodrug with increased antitumor activity in U937 leukemic cells (**1d**).

Continuing with the focus on the norbornene framework, biperiden (**1e**), an anticholinergic drug used for the treatment of Parkinson’s disease [26] that contains norbornene in its structure, was studied as a new anticancer drug for the treatment of pancreatic cancer. This molecule was able to target the protein mucosa-associated lymphoid tissue 1 (MALT1), which is frequently overexpressed in pancreatic ductal adenocarcinoma (PDAC). Biperiden reduced the cell proliferation of Panc-1 and Panc-2 cells in a time- and dose-dependent manner. In an in vivo study, the average tumor size after treatment with biperiden was reduced by 83% relative to the control in immunodeficient mice injected with Panc-1 cells. Furthermore, biperiden had tolerable side effects with minor motor side effects on mice. This norbornene-containing compound has the capacity to antagonize muscarinergic receptors, which are frequently expressed in cancer cells, responsible for cancer progression and the promotion of the proapoptotic factor BAX [27].

**Figure 2:**
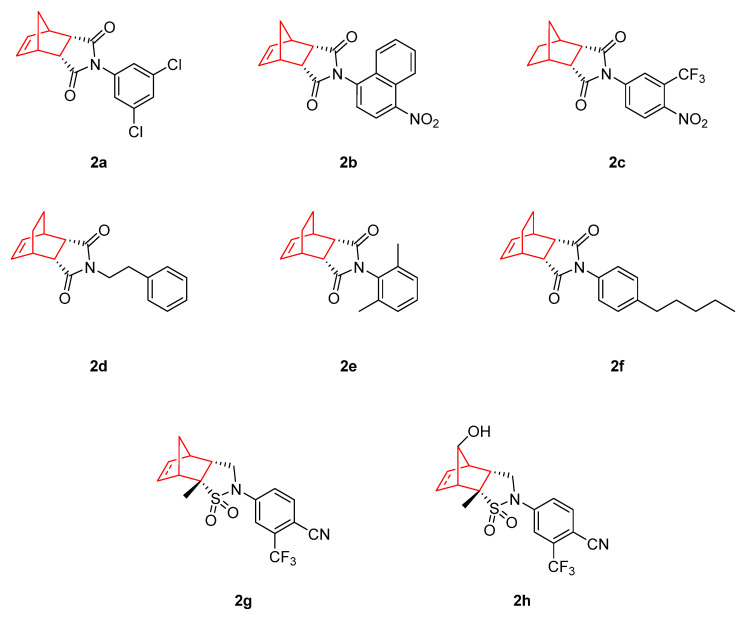
Androgen receptor (AR) antagonist with antitumor activity based on bicyclic imides containing the norbornene structure (compounds **2a–2f**) [29,30,31] and bicyclic sultams (compounds **2g–h**) [32].

Among the different tumors, prostate cancer is one of the most common cancers observed in men. Androgen deprivation therapy (ADT) has long been the treatment of choice as the backbone of all other therapies, and functions by reducing circulating androgens to castration levels and slowing the progression of the disease [28]. Figure 2 shows a range of androgen receptor (AR) agonists that include norbornene in their structure. In this context, Salvati et al. [29] presented bicyclic 1*H*-isoindole-1,3(2*H*)-dione as a potent AR antagonist (**2a**–**b**). These compounds (activities are shown in Table 1) presented similar cytotoxic profiles to bicalutamide, a clinically used antiandrogen drug for the treatment of prostate cancer, with the ability to block the AR. These analogues also demonstrated potent binding affinity (K_i_), functional antagonism (IC_50_) of the wild type (WT) AR, found in the MDA-453 cell line, and functional antagonism (IC_50_) of the mutant type (MT) T877A AR, found in the human prostate cancer cell line LNCaP. From a structural point of view, the reduction of the double bond in the norbornene moiety (**2c**) led to a significant increase in binding to the AR in MDA-MB-453 cell lines, but not significantly in LNCaP and PCa2b. In addition, considering the stereochemistry of the bicyclic structure, the *endo*-isomer showed significantly better binding and functional antagonist activity toward the WT AR compared to the *exo*-isomer [30]. Based on these findings, new compounds with the expansion of the bicyclic portion were synthesized, showing that there is not a clear correlation between the bridge expansion and the AR binding activity. Additionally, substitutions around the bicyclic portion did not generate a clear pattern in the activity of these molecules, and the activity against the AR isoforms found in the LNCaP and Pca2b cell lines was dictated by the aniline portion of the molecule, not by the bicyclic one [29]. Another study, performed on similar structures, showed that the bicyclic imides (**2d–f**) presented low IC_50_ (14, 13 and 10 µM, respectively) values in African green monkey kidney cells (Vero). This research determined that replacing the bicyclic structure with other planar structures, such as phthalimides, may provoke a decrease in the cytotoxic activity, proving that the norbornene structure is the key for the activity of these molecules. Moreover, the substituent at the *N*-position seems to be a determinant factor. These bicyclic imides exhibited marked affinity for DNA binding and significant apoptosis levels, as indicated by their percentages for inducing apoptosis in blood neutrophils after 72 h, these being 68.1, 68.0, and 70.0 % for **2d**, **2f**, and **2g**, respectively [31].

As a next step, Shan et al. [32] synthesized structurally related analogues, i.e., [2.2.1]bicyclic sultams (**2g** and **2h**), by inserting the sulfone group into the bicyclic core. These compounds displayed improved chemical stability compared to bicyclic imides, and were tested as AR antagonists. The lead compound (**2g**) was evaluated in vivo in immature rats with a daily oral dosage of 150 mg/kg for 20 days. The bicyclic sultam **2g** presented tumor stasis during the treatment, whereas bicalutamide showed only a 39% growth inhibition, and toxicity was not observed. Moreover, the structural alteration of the norbornene scaffold of compound **2g** through the hydroxylation of the C_7_ in the bicyclic bridge reduced the activity of the compound (**2h**) [32].

**Figure 3:**
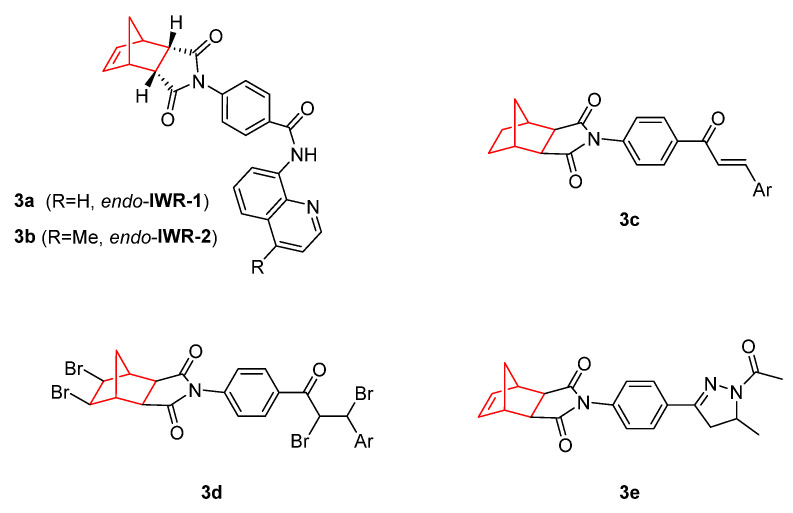
Wnt response inhibitors containing norbornene [34,35,36,37,38] (compounds **3a, 3b**). Carbonic anhydrase (CA) inhibitors containing norbornene scaffolds [39,40,41] (compounds **3c–e**).

The use of similar structures to the abovementioned to treat other tumors and explore new mechanisms of action has been performed by several research groups. Figure 3 displays several structures of inhibitors of carbonic anhydrase (CA) and the Wnt response that contain norbornene in their structure. The role of Wnt signaling in carcinogenesis has most prominently been described for colorectal cancer, but aberrant Wnt signaling is also observed in other types of cancers [33]. In this sense, compounds **3a** (*endo*-IWR-1) and **3b** (*endo*-IWR-2) have arisen as antagonists of the Wnt/β catenin pathway. They were selected from about 200,000 compounds from a synthetic chemical library through a cell-based screening strategy. *Endo*-IWR-1 and *endo*-IWR-2 suppressed Wnt signaling by stabilizing the axon destruction complex, a negative regulator of canonical Wnt signaling. The addition of *endo*-IWR-1 (**3a**) to the aquarium water of zebrafish suppressed fin regeneration after mechanical resection, but not when *exo*-IWR-1 was added. This simple and rapid assay of Wnt/β catenin pathway activity showed notable differences between *endo* and *exo* forms, one example being that *exo*-IWR-1 was 25-fold less active than *endo*-IWR-1. Accordingly, *endo*-IWR-1, but not *exo*-IWR-1, inhibited the expression of FGF20a, a gene whose expression is induced by Wnt/β catenin pathway activity [34]. Further studies on the structure–activity relationship (SAR) of these molecules showed that the saturation of the olefin on the norbornene moiety did not affect the activity of the compounds, indicating that norbornene can tolerate subtle steric perturbations [34,35].

Additional studies performed on IWR-1 confirmed that this norbornene derivative is a potent agent for colon cancer cell destruction. It inhibited epithelial mesenchymal transmission (EMT) of the colon carcinoma cell lines as well as HCT116 and HT29 cell invasion and migration. IWR-1 has also demonstrated potential to suppress tumor metastasis by inhibiting the Wnt/β-catenin pathway and survivin expression [36]. In addition, compound **3a** is also specifically cytotoxic for osteosarcoma cancer stem-like cells, inducing apoptosis of osteosarcoma spheres. In a study performed by Martins-Neves et al., it was observed that the combination of this norbornene derivative with doxorubicin provides synergistic effects by increasing cytotoxicity and substantially decreasing tumor progression [37].

Other CA inhibitors are norbornene bicyclic imides (**3c–e**). This enzyme plays a pivotal role in the treatment of diverse diseases as glaucoma, obesity, and cancer. Methanoisoindole-1,3(2*H*)-diones (**3c**) showed very high anticancer activity, with the inhibition range between 80.5–97.0%. The presence of different phenyl rings with substituents in the *ortho*-position as well as furan or pyridine resulted in the most promising derivative against the C6 cell line derived from gliocarcinoma [39]. These same authors described tetrabromo chalcone derivatives containing 4,7-methanoisoindol-1,3-dione (**3d**) that exhibited excellent inhibitory effects in the low nanomolar range, resulting in better inhibitory effects than acetazolamide (AZA), which is used in clinic as CA inhibitor [40]. Following the abovementioned investigation, other compounds structurally related to **3e** that showed antiproliferative activity, with IC_50_ values in the range of 33.4–95.0 µM, on C6 cells were reported [41].

**Figure 4:**
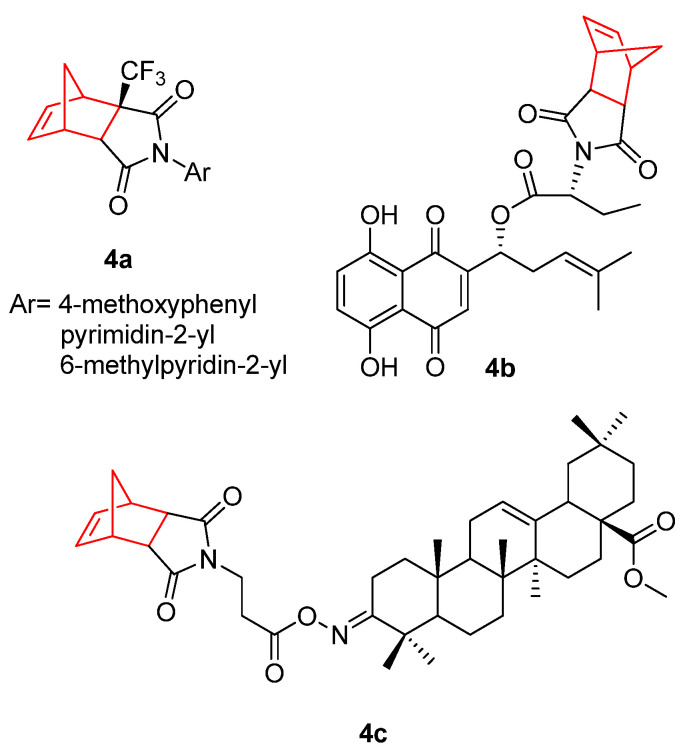
Different norbornene-containing molecules with antitumor effects [42,43,44,45,46,47,48].

Apart from the abovementioned use of norbornene bicyclic imide derivatives as agonists of AR, CA, or Wnt responses, this structure has also demonstrated antiproliferative properties against different tumor cell lines (Figure 4). In this sense, new trifluoromethyl-containing succinimides (**4a**) have been reported. The introduction of fluorine and fluorinated groups as CF_3_ in prodrugs is an important tool in medicinal chemistry [42], and the incorporation of this group into the bicyclic structure to form the *exo*-CF_3_ cycloadducts developed potent cytotoxic activity on several cancer cell lines, such as leukemia cell lines (RPMI-8226—myeloma cell line), non-small cell lung cancer cell lines (A549/ATCC—lung carcinoma epithelial cells), and SN12C renal cancer cell lines [43]. Moreover, the conjugation of the norbornene–succinimide motif with a derivative of the natural product shikonin (**4b**) displayed the strongest antiproliferative effects against HeLa, HepG2, and MCF-7 cancer cells from a library of 50 diverse compounds [44].

In the last few years, numerous triterpenoids have been studied due to their cytotoxic effects against several types of cancer. Oleanolic acid, a common triterpenoid found in olive oil, is a potent inhibitor of cellular inflammatory processes [45]. It inhibits tumor progression, invasion, and metastasis [46,47]. The antitumor activity of oleanolic acid can be improved by the addition of norbornene-2,3-dicarboximide moieties. The best example is compound **4c**. The incorporation of the norbornene-imide moiety to oleanolic acid increased the cytotoxicity of the molecule about 2.8 times in HeLa cells (4.27 µM), four times more active against KB, HepG2, and HDF cells (3.7, 4.0, and 4.4 µM, respectively), and five times more active against breast cancer cells MCF-7 (2.7 µM) in comparison with oleanolic acid alone [48].

**Figure 5:**
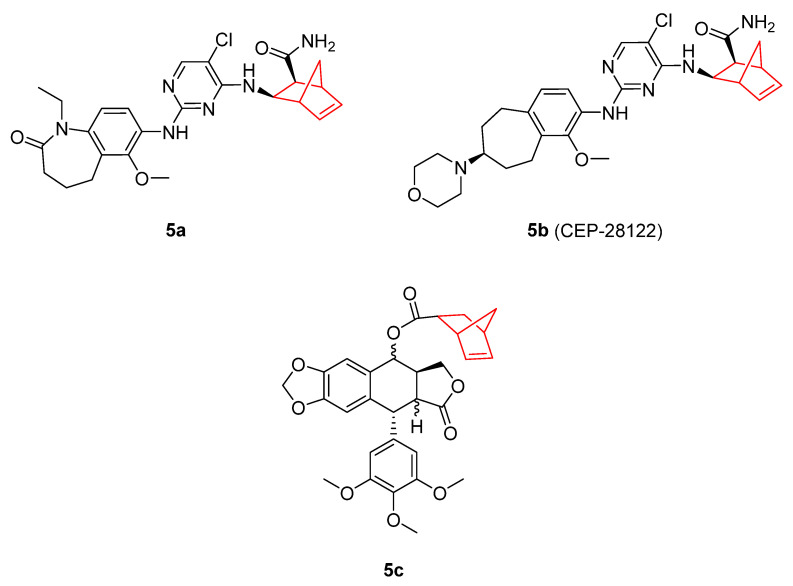
Anaplastic lymphoma kinase inhibitors containing norbornene (compounds **5a–b**) [49,50,51]. Podophyllotoxin–norbornene derivative (compound **5c**) [52,53].

Other types of norbornene derivatives with antitumoral properties, which present different chemical structures from the bicyclic imides above described, are represented in Figure 5, displaying different mechanisms for tumor regression. Anaplastic lymphoma kinase (ALK) is a kinase implicated in tumoral processes, mainly lymphomas and neuroblastomas. Compound **5a** displayed in vivo antitumor activity with the activation of the proapoptotic caspases 3/7 and selective cytotoxicity against ALK-positive ALCL cells. The antitumor activity was dose-dependent, with complete tumor regression in mice at 55 mg/kg [49]. Compound **5a** was optimized through modifications of the diaminopyrimidine moiety of the molecule, resulting in a potent orally bioavailable ALK inhibitor [50]. Following these works, these authors described a next generation of ALK inhibitors that improved these results. The preclinical candidate CEP-28122 (**5b**) presented 600-fold higher selectivity compared to the insulin receptor (InR) toward previously described ALK inhibitors, along with better absorption than **5a**. In a panel of 255 kinases, CEP-28122 achieved ≥ 90% inhibition at 1 µM for 15 kinases [51].

In addition, it is worth mentioning a research study performed to explore whether norbornene was suitable for reinforcing/modulating the antitumoral properties of well-known chemotherapeutic agents such as podophyllotoxin. Norbornene-derived podophyllotoxin carboxylate isomers 7α, 8β (**5c**) presented IC_50_ values of 5 nM for P-388, and 4 nM for A-549, HT-29, and MEL-28 cancer cell lines. These values were threefold lower than those produced by etoposide (12–24 nM), a podophyllotoxin precursor [52]. Further investigation showed an EC_50_ of 0.19 µM in human lung carcinoma A549 cell line. This compound, and similar derivatives with a ring extension in the norbornene moiety, also show a total depolymerization of the cell microtubules, such as podophyllotoxin. Ring expansion of the norbornene moiety also causes a decrease in the cytotoxic activity [53].

## 3. Camphor and Borneol Derivatives

**Figure 6:**
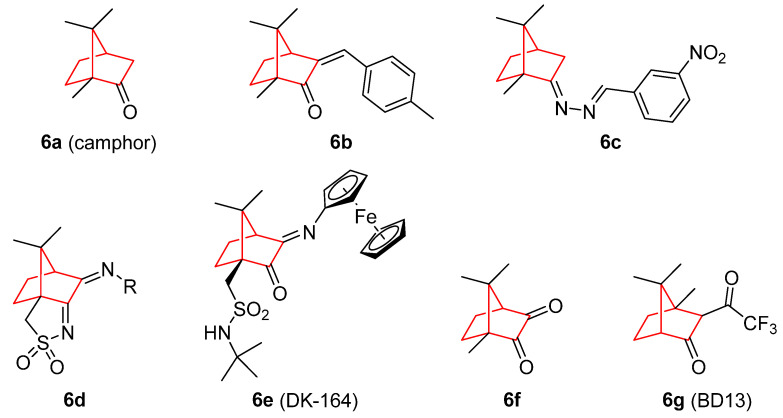
Camphor [54,55,56,57] and several other derivatives with antitumoral activity [58,59,60,61,62,63,64].

Camphor (1,7,7-trimethylbicyclo[2.2.1]heptan-2-one, **6a**) (Figure 6) is another interesting norbornene-related structure. It is a natural product used since antiquity in a wide range of biological applications, anticancer properties being one of the most representative. Camphor is present in *Salvia libanotica*, and its essential oil possess antitumor properties against two isogenic human colon cancer cell lines HCT-116 (p53^+/+^ and p53^−/−^), and has no effect on the growth of normal human intestinal cell line FHs74Int [54]. These properties have been corroborated for other camphor-containing plant extracts, such as *Cinnamomum camphora* [55] in a mouse model of keratinocyte-derived skin cancer; *Chiliadenus antiatlanticus*, which showed significant toxicity towards HepG2 and melanoma cells [56]; and *Thymus algeriensis* activity against the colon and hepatic cancer cell line HCT116 [57].

In this context, 4-methylbenzylidenecamphor (**6b**) was able to activate the estrogen receptors (ER), ERα and ERβ, in an animal model [58]. Other modifications to this structure have implicated the formation of hydrazones, imines, nitroimines (**6c**) [59], and sulfonylimines (**6d**) [60]. Silver complexes with this sulfonylimine displayed IC_50_ values by, at least, one order of magnitude lower than cisplatin, a well-known chemotherapeutic drug. The combination of camphorsulfonamides with ferrocene (**6e**) showed potent cytotoxicity values in breast cancer cells (MCF-7 and MDA-MB-231), with cell cycle arrest in G1 phase [61], and in lung cancer cells (A549 and H1299) [62].

An important derivative of camphor is camphorquinone (**6f**), which inhibited the cell growth in L5178/TK^+/−^ in a concentration-dependent manner, and is able to trigger high intracellular reactive oxygen species/reactive nitrogen species (ROS/RNS) generation [63]. In addition, Nakano et al. [64] synthesized a variety of β-diketones, some of which derived from camphor. In this regard, the analogue BD13 (**6g**) showed cytotoxicity towards HSC-2, HSC-3, HSG, and HL-60 cells, presenting great tumor-specific cytotoxicity (SI = 4.3) without apoptosis induction.

**Figure 7:**
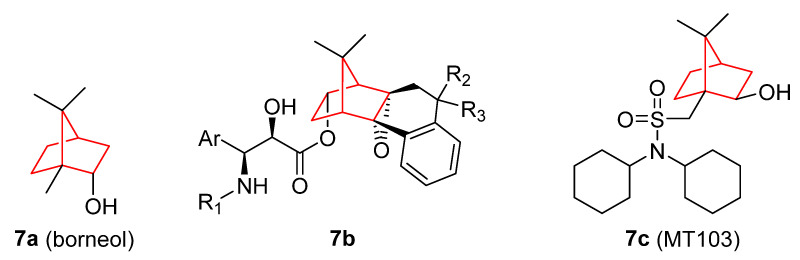
Borneol derivatives [65,66,67,68,69,70,71,72,73,74,75,76,77].

Borneol (**7a**) is a natural compound that can be easily derived from camphor, and it is present in the essential oil of several plants, such as *Tanacetum gracile*. Borneol-containing essential oils have been proposed as mitochondrial-dependent apoptosis inductors in human leukemia HL-60 cells [65]. Natural borneol has been identified as a potential chemosensitizer in treating human cancers. In melanoma cells (A375), borneol sensitized these cells to apoptosis induced by curcumin through the activation of ROS-mediated DNA damage, upregulation of phosphorylated JNK, and downregulation of phosphorylated ERK and Akt [66]. Similar behavior was observed with bisdemethoxycurcumin in HepG2 cells [68,69]. The co-treatment of borneol with temozolomide, a chemotherapeutic agent used for treating human glioma, provoked DNA damage through ROS induction and mitochondrial dysfunction [69]. It also synergized with paclitaxel, another chemotherapy drug, consequently inhibiting the survival of human esophageal squamous cell carcinoma through apoptosis induction [70], and increased the anticancer efficacy of doxorubicin against human glioma in an in vivo model [71]. In addition, borneol also augmented the therapeutic effects of doxorubicin in vitro and in vivo through TRPM8-regulated calcium mobilization in A549 human lung adenocarcinoma, probably by the suppression of the biological function of multidrug resistance protein P-gp, and enhancement of cellular uptake of doxorubicin [72]. Another interesting property of borneol is its ability to selectively open the blood–tumor barrier (BTB), and consequently increasing the permeability [73], possibly through the activation of phosphorylation of ERK, and further downregulating reversibly the expression of associated proteins [74]. This favors the decrease in some limitations of the chemotherapeutic agents, such as cisplatin, due to difficulties in penetrating the BTB [75].

Some structural modifications have been introduced into the borneol structure (Figure 7), such as the esterification of the hydroxyl group (**7b**). The new compounds generated can act as inhibitors of microtubule depolymerization with higher potency than paclitaxel [76]. Moreover, the derivatization of the borneol isomer isoborneol with sulfonamide moiety led to the MT103 (**7c**) compound, which demonstrated the capacity to inhibit tumor growth of a great variety of the NCI-60 cancer cell line panel via the induction of apoptosis [77].

## 4. Metal Complexes with Norbornene and Norbornene-Related Compounds

**Figure 8:**
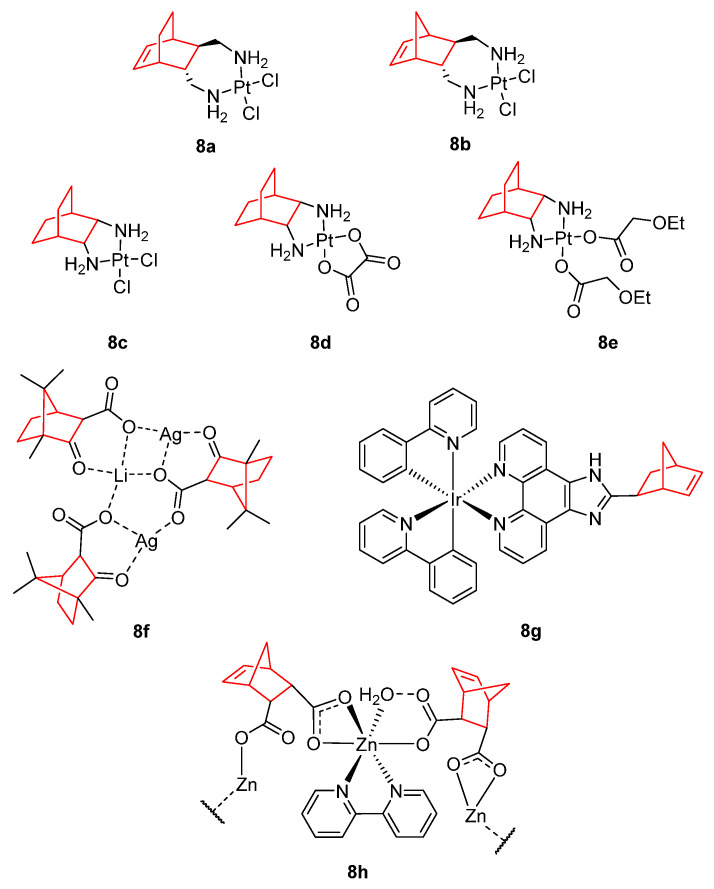
Metal complexes containing a norbornene scaffold and its bicyclo[2.2.2]octane analogue [81,82,83,84,85,86].

Metal complexes (Figure 8), mainly platinum, have been used in chemotherapy for cancer patients with multiple types of solid tumors. Since the Food Drug Administration (FDA) approved the use of cisplatin for cancer treatment, the search for new platinum complexes as antitumor drugs has attracted extensive research [78]. Currently, cisplatin, carboplatin, and oxaliplatin are the most common platinum complexes employed in clinical practice [79,80,81]. Considering norbornene as a privileged scaffold and the importance of platinum in cancer, De Mier-Vinué et al. [81] reported a chemical library of six platinum (II) complexes possessing 1,2-bis(aminomethyl)carbobicyclic ligands that exhibited cytotoxic activity when tested against cisplatin resistant cell lines. The increase in steric hindrance produced an increase in the biological activity. Compounds with an ethylene bridge (**8a**) afforded better results than those with a methylene bridge (**8b**) [81].

With the aim of improving the pharmacological properties of platinum complexes, different bicyclo[2.2.2]octane ligands have been incorporated to these metal complexes (**8c**–**e**) and tested by Liu et al. [82,83]. Metal complex **8e** showed potent cytotoxicity against HCT-116, HepG2, and A549 cancer cell lines (see Table 2). Its cytotoxic activity against HCT-116 colorectal cancer cells was comparable to the observed values for oxaliplatin and higher that values derived from cisplatin and carboplatin data. Furthermore, **8c** also showed lower toxicity (IC_50_ = 44.3 µM) than cisplatin and oxaliplatin (IC_50_ = 25.3 and 31.8 µM, respectively) in HUVECs (human umbilical vein endothelial cells), indicating a greater selectivity. The introduction of the bicyclic framework increased the antitumor activity of the platinum complexes due to its steric hindrance. Docking and agarose gel electrophoresis studies revealed that the interaction of **8c** with the DNA was similar to that of oxaliplatin. Western blot indicated mitochondrial-dependent apoptosis pathway, stronger than oxaliplatin [83].

Derived from camphorquinone, new camphor carboxylate (**8f**) and carboxamide silver complexes have been reported that are more active than cisplatin, with high selectivity towards A2780 ovarian cancer cell line [84].

In recent years, organometallic iridium complexes have arisen as an alternative to platinum-based drugs owing to their higher antineoplastic activity, lower side effects, photochemical activity, and redox properties. The norbornene-containing ligand 2-bicyclo[2.2.1]hept-5-en-yl-1H-1,3,7,8-tetraazacyclopenta[l]phenanthrene (BTCP) was prepared by reaction of 1,10-phenanthroline-5,6-dione with 5-norbornene-2-carboxaldehyde [85]. BTCP presented no antitumor effect in isolation, but the reaction with [Ir(ppy)_2_Cl_2_]_2_ to form **8g** improved the cytotoxic effects of [Ir(ppy)_2_Cl_2_]_2_. Derivative **8g** showed IC_50_ values of 3.9, 5.4 and 4.1 µM in SGC-7901, MG-63 and SiHa cells, respectively. Additionally, **8g** increased the ROS levels, induced a decrease in the mitochondrial membrane potential, and inhibited cell growth at G0/G1 phase of the cell cycle in human gastric cancer cell line (SGC-7901).

The use of Zn(II) instead of Pt(II) is another potential approach for cancer therapy considering that it is essential for all forms of life and it is critical for numerous cell processes. In this sense, Zn(II) complexes with endo-norbornene-cis-5,6-dicarboxylic acid (**8h**) have been demonstrated to affect viability of HeLa and KBr cell lines and to induce apoptosis via DNA-cleavage activity [86].

## 5. 7 Oxanorbornene Derivatives

Heteroatom introduction on methylene bridge in norbornene structure is a clear modification strategy due to the readiness of the DA reaction with the corresponding heterocycle. However, this modification is not as easy in practice because of the high aromaticity of heterocycles such as furan, thiophene, and pyrrole, reducing the scope of this strategy [87]. Furan is the most reactive heterocyclic diene, and its DA adducts have shown biological activities against cancer; thus, these derivatives are the main object of discussion in this section.

Norcantharidin (**9a**), the simplest synthetically available 7-oxanorbornene derived from the DA reaction of maleic anhydride and furan, is the demethylated analogue of naturally occurring cantharidin (**9g**). Norcantharidin inhibited the proliferation of many tumor cells both in vitro and in vivo, including cervical, hepatoma, ovarian, laryngocarcinoma, colon, breast, prostate, osteocarcinoma, and leukemia [88,89,90,91,92,93]. Different mechanisms of action have been described for its antitumoral activity from apoptosis to autophagy or cell proliferation inhibition [94]. In general, norcantharidin induced mitotic arrest of the G2/M phase in several cancer cell lines [95,96,97,98,99]. In HT-29 and HCT-116 colon cancer cell lines, norcantharidin induced a G2/M phase cell population accumulation and cell death, affecting apoptosis related to signaling proteins in a dose-dependent manner, and resulting in an effect more potent than gefitinib. Norcantharidin also inhibited HT-29 colorectal cancer cells growth through a decrease in the ανβ6 expression and inhibited ERK phosphorylation. In addition, this derivative has been studied in other tumoral cell lines, and different mechanisms of action have been proposed. In hepatocellular carcinoma cells, results revealed an increase in the expression of FAM46C with a decrease in Ras, MEK1/2, and ERK1/2 phosphorylation [100,101]. In nasopharyngeal carcinoma, 9a inhibited the proliferation and induced apoptosis with activation of caspases 3, 8, and 9, and a significant reduction in B-cell lymphoma-extra large (Bcl-XL) expression [102]. In osteosarcoma, norcantharidin provoked G2/M phase arrest and cell apoptosis with a decrease in the phosphorylation of Akt [103]. In triple-negative breast cancer, it induced cell senescence and cell cycle arrest, which was accompanied by a decline in phosphorylated Akt and ERK1/2 [104]. In non-small cell lung cancer, 9a restrained cell progression via the regulation of the AMPK/mTOR-mediated autophagy pathway [105]. In human oral squamous cell carcinoma, norcantharidin 9a activated the p38 MAPK pathway [106]. In renal cell carcinoma it induced apoptosis, G2/M phase cell cycle arrest accomplished with decrease in the expression of procaspases 3 and 9, cyclin B1, and phosphorylation of ERK and JNK, but not p-38 MAPK [107].

These results have encouraged researchers to construct new structures combining the norcantharidin scaffold with other fragments. An interesting structural modification is the preparation of N-substituted norcantharimide compounds such as the *N*-farnesyloxy and *N*-farnesyl norcantharimides (**9b** and **9c**, respectively). These derivatives exhibited the highest cytotoxicity, and antiproliferative and apoptotic effects against HepG2 human hepatoma cell lines without cytotoxic effects on murine embryonic liver BNL CL.2 cells [108]. In addition, they proved to be safer anticancer drugs than norcantharidin [109]. Another study based on norcantharidin derivatives was developed by Cheng et al. [110]. They reported new unsaturated norcantharimide dimers (**9d**), and concluded that the in vitro antitumor properties against Hep3B HCC and the KG1a AML cell lines depended mainly on the length of link chains. In this sense, norcantharidin inspired tetrahydroepoxyindole carboxamides (**9e**) displayed superior activity against breast cancer line MCF-7 than cisplatin [111]. Wang et al. [112] reported new conjugates constructed by assembling different monoacid monoesters of norcantharidin derivatives and camptothecin using hydroxyl acetic acid as linker (**9f**). The new molecules preserved similar activities against HepG2, BGC803, SW480, and PANC-1 cell lines in vitro compared with positive control cantharidin [113]. Another simpler approach for norcantharidin modification is salt formation. Zhao et al. [114] described a new series of norcantharidic acid mixed salts (Na, K, Mg, and Ba) with promising antitumor activity against both liver and colon cancer cell lines in vitro.

It is remarkable that norcantharidin has been combined with classic chemotherapeutic agents. For example, the combination of norcantharidin and crizotinib, a mesenchymal epithelial transition factor (c-Met) inhibitor, was more effective than norcantharidin alone in restraining both proliferation in vitro and tumor growth in vivo in hepatocellular carcinoma [115]. On the other hand, co-administration with diamine dichloroplatinum had an additive effect with significant suppressions of tumor growth and metastasis [116]. In addition, this complex provoked an increase in nuclear condensation and mitochondrial membrane depolarization, along with apoptosis induction in hepatocellular carcinoma when combined with a glycolytic inhibitor, such as 2-deoxy-D-glucose [117]. In a study carried out on prostate cancer, the combination of norcantharidin and paclitaxel inhibited cell proliferation, enhanced G2/M phase arrest, induced cell death, ER stress, and decreased the mRNA expression of SIRT7 [118].

**Figure 9:**
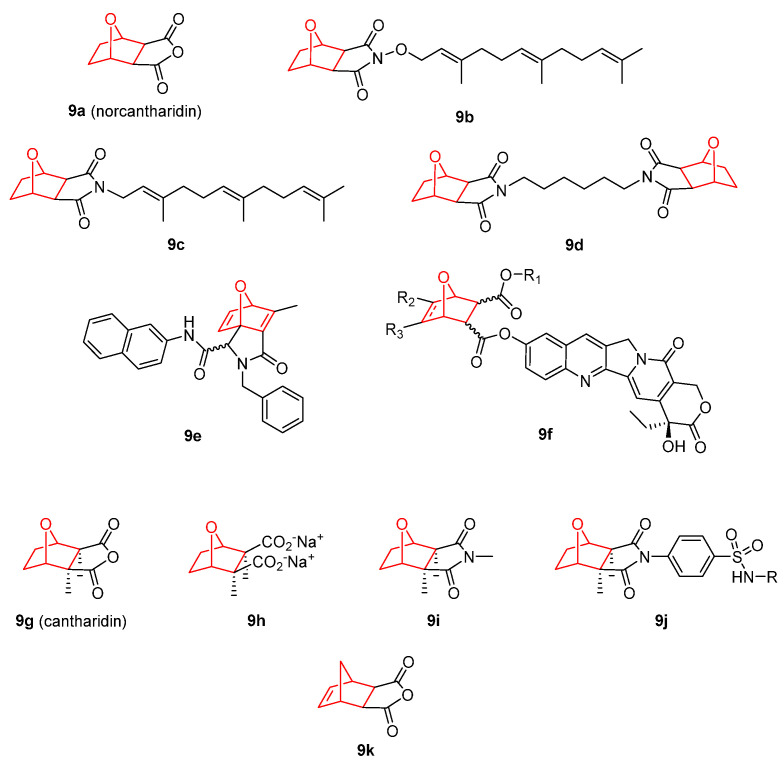
Norcantharidin [88,89,90,91,92,93,94,95,96,97,98,99,100,101,102,103,104,105,106,107,108,109,110,111,112,113,114], cantharidin [119,120,121,122,123,124,125,126,127,128,129,130,131], and related molecules.

As mentioned above, the natural precursor of norcantharidin is cantharidin (**9g**). This 7-oxanorbornene scaffold-containing compound also presents anticancer properties, and is the major bioactive component that can be extracted from dried Chinese blister beetle *Mylabris phalerata* [119]. Numerous reports can be found in the literature regarding the cytotoxic activity of cantharidin and its analogues against different types of cancer, such as breast [120], pancreas [121], colorectal [122], and lung [123]. From all the mechanisms of action proposed for cantharidin derivatives, apoptosis, and DNA damage through the inhibition of protein phosphatases are the most commonly accepted. The growing attention of scientific community toward cantharidin has led to the proposal of several modifications. Among all the derivatizations, salt formation has been one of the most interesting due its simplicity. For example, sodium cantharidinate (Figure 9, **9h**) induced apoptosis and DNA fragmentation of pancreatic cancer cells (PANC-1) with perturbation in p53 signaling pathway [124]. Kian’s group tested this salt against breast cancer and detected apoptosis and cell cycle arrest with possible implication of p53 [125]. Moreover, Chen et al. [126] reported a study related to the synergistic effect between cisplatin and sodium cantharidinate for cervical cancer treatment. On the other hand, the acid form (cantharidic acid) was able to induce apoptosis through p-38 upregulation [127].

Another subtle derivatization proposed for cantharidin is the synthesis of imides. The new designed analogue methyl cantharidimide (Figure 9, **9i**) was effective against endocervical adenocarcinoma cell line KB-C2 overexpressing ABCB1 and against human SDHB knockout HEK-293 cell line, which overexpresses ABCG2, as well as cisplatin-resistant cells, such as KCB-4 [128]. Other relevant derivatives are the cantharidin–sulfanilamides (Figure 9, **9j**) that exhibited anti-HL-60 and anti-Hep3B cell activities [129].

On occasion, the type of heteroatoms in the bridge substitution is a key point in the cytotoxicity of the molecule. The drawbacks of cantharidin and norcantharidin are their cytotoxicity against normal cells [130]. Compound 9k, a norbornene analogue of dehydro-norcantharidine in which the oxygen in the bridge has been replaced by a methylene, showed IC_50_ values of 62 µM (HepG2) and 152 µM (SK-Hep1), similar to norcantharidin (74 and 126 µM, respectively). Nevertheless, the toxicity in rat hepatocytes is clearly lower for **9k** (IC_50_ = 253 µM) than for norcantharidin (IC_50_ = 75 µM). Additionally, hepatocellular carcinoma cells HepG2 and SK-Hep1 treated with cantharidin (**9g**), norcantharidin (**9a**), and **9k** showed chromatin and nucleus condensation, a signal of apoptosis. Rat hepatocytes treated with cantharidin and norcantharidin presented the same results, but not for 10 h, where chromatin was homogenously distributed within the nuclei [131].

**Figure 10:**
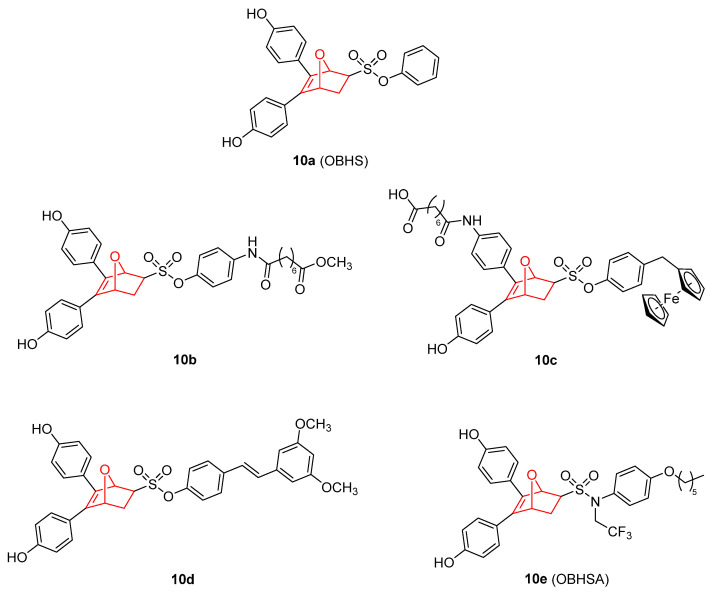
OBHS derivatives with estrogen receptor agonist activity [133,134,135,136,137,138].

Other functionalization in the parent diene derived from furan produced sulfonamide and sulfonate derivatives (compounds of Figure 10). These molecules are ligands for the ERα studied for the treatment of breast cancer. Most of these compounds are synthesized by a DA reaction of substituted furans with a variety of dienophiles in a simple manner [132]. OBHS (10a) is an ERα ligand, which is effective for the prevention and treatment of estrogen-dependent endometriosis in vivo [133]. OBHS can arrest the cell cycle of MCF-7 breast cancer cells at S phase, and induce apoptosis along with the decreased protein expression of antiapoptotic proteins BCL-2 and MCL-1. In combination with olaparib and doxorubicin, OBHS delays the tumor progression in vivo and avoids the lethal effect of these anticancer drugs. OBHS also decrease the expression of ERα and knockdown of Erα-sensitive breast cancer cells to olaparib and doxorubicin [134].

Taking into account the importance of the molecular hybridization strategy in the design of drugs, new conjugated hybrids of OBHS have been reported. Among the new analogues, hybrids with histone deacetylase inhibitors (HDAC) [135], such as vorinostat, (compound **10b**) have emerged for breast cancer treatment. The conjugation of these two complementary bioactive units demonstrated higher beneficial effects and fewer side effects than single-target agents via simultaneously modulating multiple targets and circumventing differences in pharmacokinetic profiles. In order to extend the therapeutic effectivity of OBHS–HDAC conjugates above for both ER(+) and ER(−) breast cancer cells, Li et al. [136] reported a new family of ferrocene complexes (termed FcOBHS–HDACi conjugate) based on the OBHS–HDAC core scaffold (**10c**). These compounds presented significant antiproliferative effects on ER(−) MDA-MB-231 and ER(+) MCF-7 breast cancer cells and potent HDAC inhibition [136]. Another interesting example of new conjugates is compound **10d**, that results from the attachment of resveratrol moiety into the OBHS scaffold [137]. This derivative acted as a good ER ligand with excellent ERα antagonistic activity, potent anti-inflammatory activity, and exhibited higher activity than tamoxifen in vivo. In addition, when the sulfonate group on OBHS was replaced with sulfonamide, the resulting OBH-sulfonamide **10e** (OBHSA) exerted full antagonist activity with efficacy in ERα-positive MCF-7 comparable to that of fulvestrant [138].

**Figure 11:**
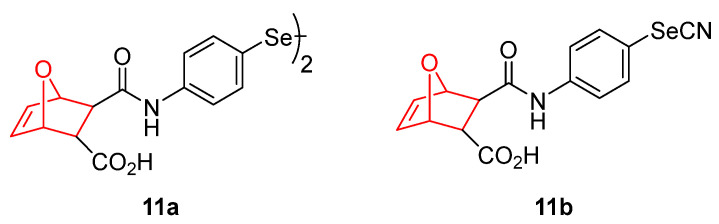
Selenium containing oxabicyclic derivatives [142].

Another attractive modification for molecular hybridization in cancer therapy is the inclusion of selenium functional groups [139]. Based on the DA reaction, our research team designed and synthesized a novel series of oxabicycle[2.2.1]selenocyanates and diselenides. Among them, derivatives **11a** and **11b** (Figure 11) showed potent and broad antitumor activity against different cancer cell lines. The breast carcinoma cell line MCF-7 was the most sensitive, showing TGI values of 9.2 µM and 11.6 µM for **11a** and **11b**, respectively. Despite selenocyanates [140] and diselenides [141] having demonstrated their potential antitumor activity, the incorporation of this bicycle core via DA cycloaddition enhanced the antitumor effect in comparison to other analogues containing different scaffolds. Compound **11b** arrested the cell cycle at phase S and triggers caspase-independent programed cell death. The increased levels of beclin1 and LC3-IIB and reduced levels of SQSTM1/p62 in MCF-7 treated cells suggest autophagy as the cell death mechanism [142].

## 6. Drug Delivery Systems Containing Norbornene and Related Scaffolds in the Treatment of Cancer

Drug delivery systems (DDS) have gained attention because they can modify the chemical absorption, distribution, metabolism, excretion, and toxicity (ADMET) profiles of pharmaceutical entities. DDS not only can increase the water solubility of drugs and protect them within the bloodstream, which improves the pharmacokinetic and pharmacological properties of the drugs, they can also deliver them to a target tissue or specific cell, limiting drug accumulation in non-targeted organs, which reduces their side effects and leads to an improvement in the therapeutic efficacy of antitumoral drugs [143]. Due to their beneficial properties, the development of DDS loading norbornene and other derivatives has also been explored in the treatment of cancer either using passive or active targeting. In this context, studies suggest that the norbornene derivative borneol enhances drug delivery across different physiological barriers such as the blood–brain barrier (BBB) [144], as mentioned before. Because of its property, different DDS have been designed to treat glioma or glioblastoma. In detail, carmustine-loaded micelles functionalized with borneol and a peptide (pep-1) evaluated in a Luc-BT325 glioma tumor-bearing nude mice model exhibited a strong inhibition of tumor growth, long survival period, and low systemic toxicity, with a long retention time in brain tissues [145]. In the same line, doxorubicin-loaded nanomicelles combined with borneol inhibited the tumor growth and metastasis of glioblastoma in vivo [146]. On the other hand, norcantharidin-loaded multifunctional metal–organic framework incorporated in a thermosensitive gel was efficiently delivered to Hepa1-6 liver cancer cells, decreasing the toxicity and increasing the bioavailability of the drug in vitro, which represents an effective method for passive targeting therapy of liver tumors [147]. Studies carried out with norcantharidin conjugated to carboxymethyl chitosan showed inhibition of migration of lung carcinoma tumor cells both in vitro and in vivo in a dose-dependent manner. The enhanced antitumor effects were confirmed by inhibiting the growth of solid tumors and extending the survival in a metastasis mice model [148]. Norcantharidin was also loaded in a poly(D,L-lactide)-β-poly(ethyleneglycol)-β-poly(D,L-lactide) thermosensitive hydrogel (13% *w*/*w*) for the treatment of primary hepatocellular carcinoma. After being intratumorally injected, the hydrogel was able to prolong the drug retention time in tumor compared to the free drug solution. The in vivo efficacy also demonstrated its capability to improve the curative effect of the drug and reduce its toxicity [149]. Furthermore, in vivo studies using nanoparticles of Zn(II) coordination polymers containing norcantharidin as ligands confirmed high efficacy in the inhibition of the growth of Hep3B tumors and relatively few side effects compared with the free drug [150]. On the other hand, a diacid metabolite of norcantharidin combined with ABT-737 and incorporated in silica nanoparticles coated with folate acid (FA-LB-CHMSN), with the aim of targeting the folate receptor, presented a synergistic effect, which resulted in tumor cell apoptosis and, remarkably, tumor inhibition in the H22 tumor-bearing mice model [151]. Moreover, the antitumoral effect of different DDS containing the derivative cantharidin have been assayed either in vitro or in vivo [152,153,154]. In fact, cantharidin encapsulated in nanostructured lipid carriers decorated with hyaluronic acid coupled with mPEG-NH_2_ reduced drug toxicity and increased in vivo efficacy with a 65.96% of tumor inhibition rate in a mouse model of human hepatoma using SMMC-7721 cells [155]. Finally, nanomedicines based on ring-opening metathesis polymerization under the norbornene backbone containing antitumoral drugs have appeared as a versatile platform for drug delivery and image-guided diagnostics, playing a crucial role in the field of cancer therapy [156,157].

## 7. Conclusions

Norbornene derivatives are undoubtedly among the DA compounds with outstanding potential in cancer due to diverse therapeutic benefits, as demonstrated by several research groups in recent years. Indeed, since the first observation of the anticancer effect of this structure in 1994, growing attention has been given to the use of norbornene-derived compounds as a mono- or adjuvant therapy with anticancer drugs such as paclitaxel or doxorubicin.

Additionally, there is a remarkably broad spectrum of anticancer properties of these structures towards several types of cancer, including renal, sarcoma, pancreatic, kidney, melanoma, liver, colon, leukemia, lung, glioma, and breast cancers. The targets analyzed for diverse norbornene based analogues included ER and AR, CA inhibition, apoptosis modulation, Beclin1, LC3-IIB, CDK2, BAX, Bcl2, EGFR, and VEGFR-2.

This review also summarizes how structural modifications of the bicyclic ring using different functional groups can increase their efficacy. The decoration at various positions of norbornene scaffold with different groups such as alkyl, aryl, or heteroaryl rings, sulfone, imines, and esters played a key role in governing their ability to interact with various biological targets, making them an attractive scaffold in the field of drug discovery. In order to develop novel, safer, and more effective therapeutic candidates, molecular hybridization with other pharmacophores, the use of prodrugs, the formation of metal complexes, and development of new drug delivery systems is suggested.

In summary, norbornene is a versatile nucleus in the field of medicinal chemistry, and could serve as a future therapeutic scaffold in the development of different biological active agents by modulation of its structure. Forthcoming years should bring progress in the elucidation of the activities, mechanisms of action, and efficacy of these derivatives that will provide a solid support for further in vitro and in vivo research.

## Figures and Tables

**Table 1:** Binding (K_i_) and IC_50_ values for different cancer cell lines produced by androgen receptor (AR) antagonist containing norbornene [29,30,32].

	MDA-MB-453 K_i_, nM	MDA-MB-453 IC_50_, nM	LNCaP K_i_, nM	LNCaP IC_50_, nM	PCa2b IC_50_, nM
Bicalutamide	64	173	35	400	725
**2a**	158	307	6	41	180
**2b**	1	>5	0.5	Ag *	Ag *
**2c**	5	12	2	Ag *	Ag *
**2g**	2	30	ND	ND	ND

* Ag (agonist): a compound with the ability to activate AR in the absence of dihydrotestosterone (DHT).

**Table 2:** IC_50_ of different platinum containing bicycles [82,83].

	IC_50_ (µM)		
	**HCT-116**	**HepG2**	**A549**
**8c**	4.45 ± 0.33 [83]	10.74 ± 1.05 [83]	7.72 ± 0.64 [83]
**8d**	9.69 ± 0.91 [83]	7.86 ± 0.61 [83]	46.43 ± 3.86 [83]
**8e**	9.14 ± 0.27 [82]	16.70 ± 0.48 [82]	57.82 ± 2.17 [82]
Cisplatin	6.50 ± 0.47	3.65 ± 0.31	3.86 ± 0.28
Carboplatin	44.53 ± 4.64	39.08 ± 2.95	55.20 ± 4.76
Oxaliplatin	3.28 ± 0.34	12.80 ± 1.28	8.95 ± 0.78

## Data Availability

Not applicable.

## Abbreviations

ABCB1	ABC transporters multidrug resistance 1
ABCG2	ABC transporters
ADMET	Chemical absorption, distribution, metabolism, excretion, and toxicity
ADT	Androgen deprivation therapy
Ag	Agonist
Akt	Serine/threonine kinase
ALK	Anaplastic lymphoma kinase
AMPK/mTOR	AMP-activated protein kinase/mechanistic target of rapamycin kinase
APC	Adenomatous polyposis coli gen
AR	Androgen receptor
AZA	Acetazolamide
BAX	BCL2 associated X protein
BBB	Blood–brain barrier
Bcl-XL	B-cell lymphoma-extra large
BTB	Blood–tumor barrier
BCL-2	B-cell lymphoma 2
BTCP	2-bicyclo[2.2.1]hept-5-enyl-1H-1,3,7,8-tetraazacyclopenta[l]phenanthrene
CA	Carbonic anhydrase
cAMP	Cyclic adenosine monophosphate
CDK2	Cyclin dependent kinase 2
c-Met	Mesenchymal epithelial transition factor
DA	Diels–Alder
DDS	Drug delivery systems
DNA	Deoxyribonucleic acid
EC_50_	Half maximal effective concentration
EGFR	Epidermal growth factor receptor
EMT	Epithelial mesenchymal transmission
ER	Estrogen receptors
ERK	Estrogen receptor kinase
FAM46C	Non-canonical poly(A) polymerase protein
FDA	Food drug administration
HDAC	Histone deacetylase
HUVEC	Human Umbilical Vein Endothelial Cell
IARC	International Agency for Research on Cancer
IC_50_	Half maximal inhibitory concentration
InR	Insulin receptor
JNK	c-Jun N-terminal kinase
Ki	Inhibitory constant
LC3-IIB	Microtubule-associated protein 1A/1B-light chain 3-phosphatidylethanolamine conjugate B
MALT1	Mucosa-associated lymphoid tissue lymphoma translocation protein 1
MAP	Mitogen-activated protein
MCL-1	Myeloid cell leukemia-1
MEK1/2	Mitogen-activated protein kinase kinases 1/2
MOF	Metal–organic framework
MT	Mutant type
PDAC	Pancreatic ductal adenocarcinoma
P-gp	MDR1/P-glycoprotein
Ras	Rat sarcoma virus
RNS	Reactive nitrogen species
ROS	Reactive oxygen species
SAR	Structure–activity relationship
SDHB	Succinate dehydrogenase complex subunit b
SI	Selectivity Index
SIRT7	Sirtuin protein 7
SQSTM1/p62	Sequestosome 1
TGI	Total growth inhibition
TNF	Tumor necrosis factor
TRPM8	Transient receptor potential cation channel subfamily M member 8
VEGFR-2	Vascular endothelial growth factor receptor 2
WT	Wild type
